# Antarctic Fish Antimicrobial Peptides Active Against Bacterial and Viral Pathogens of Aquacultural Importance

**DOI:** 10.3390/antibiotics15060527

**Published:** 2026-05-22

**Authors:** Federica Massaro, Luana Cortinovis, Romy Lucon Xiccato, Eleonora Fiocchi, Amedeo Manfrin, Anna Rita Taddei, Paolo Roberto Saraceni, Fernando Porcelli, Anna Toffan, Francesco Buonocore

**Affiliations:** 1 Department for Innovation in Biological, Agrofood and Forest Systems, University of Tuscia, 01100 Viterbo, Italy; federica.massaro@unitus.it (F.M.); porcelli@unitus.it (F.P.); 2 National Reference Centre for Fish, Shellfish and Molluscs Diseases, Istituto Zooprofilattico Sperimentale delle Venezie, Legnaro, 35020 Padova, Italy; lcortinovis@izsvenezie.it (L.C.); rluconxi@izsvenezie.it (R.L.X.); efiocchi@izsvenezie.it (E.F.); amanfrin@izsvenezie.it (A.M.); atoffan@izsvenezie.it (A.T.); 3 Department of Agricultural and Food Sciences (DISTAL), Alma Mater Studiorum, University of Bologna, Viale Fanin 44, 40127 Bologna, Italy; 4 Center of Large Equipments, Section of Electron Microscopy, University of Tuscia, Largo dell’Università snc, 01100 Viterbo, Italy; artaddei@unitus.it; 5 Italian National Agency for New Technologies, Energy and Sustainable Development (ENEA), Department of Sustainability, 00123 Rome, Italy; paolo.saraceni@enea.it

**Keywords:** antimicrobial peptides, antibiotic-resistance, aquaculture, microbial infections, piscidins, immunomodulatory properties

## Abstract

**Background/Objectives:** The aquaculture industry represents a fundamental food sector. One of the main limiting factors for this sector is related to bacterial diseases, for which antibiotics have been widely used worldwide for decades. In recent years, a more conscious approach to the use of antimicrobials within the framework of the One Health approach has increased the need for alternatives capable of helping with disease management while avoiding the onset of antimicrobial resistance phenomena. Antimicrobial peptides, which have a broad spectrum of action against pathogens, are a promising solution. **Methods:** In this work, we investigated the capability of three peptides (Trematocine, Chionodracine, and Cnd-m3) isolated from Antarctic fish to target bacterial and viral pathogens affecting aquaculture. Successively, we investigated their cytotoxicity versus a continuous embryonic cell line (DLEC) derived from European sea bass and their haemolytic activity against fish erythrocytes. Moreover, we evaluated their immunomodulatory effect. **Results:** Regarding antibacterial properties, Cnd-m3 was identified as the best peptide, demonstrating good bactericidal and bacteriostatic activity against various bacterial strains, including *Lactococcus garvieae*. Concerning this bacterium, ANS permeability assays showed that the Cnd-m3 peptide has a great ability to interact with its outer membrane, while TEM analysis revealed that the peptide, after destabilization of the cell membrane, interacts with nucleic structures. Considering the antiviral activity, Trematocine was effective against two tested pathogenic enveloped viruses. Moreover, the toxicity of Trematocine and Cnd-m3 was evaluated by investigating their cytotoxicity against a cell line derived from *Dicentrarchus labrax* and haemolysis against sea bass erythrocytes. Both revealed good selectivity towards pathogens at the lowest concentration. Finally, Cnd-m3 manifested light *in vitro* immunomodulatory properties. **Conclusions:** Overall, these data provide a solid basis for future studies assessing the potential applications of two of the tested peptides in aquaculture.

## 1. Introduction

The rapid growth of the human population has led to increased demand for easily accessible alimentary resources. In this context, the aquaculture sector, defined as “farming of aquatic organisms, including finfish and shellfish, by individuals, groups or corporations using interventions that enhance production” [1], represents a fundamental source for maintaining food supplies [2], becoming one of the main productive food sectors.

In particular, per capita consumption of seafood has increased from an average of 9.9 kg per person in the 1960s to 20.5 kg in 2019. Moreover, this trend is expected to continue, with a further increase of 15% predicted by 2030 [3,4]. However, it faces many challenges: aquaculture uses intensive methods to produce large quantities of food in small spaces, and this determines a series of complications [4,5]. These include the eutrophication of water in areas close to farms due to high levels of nitrogen and phosphorus released from fish metabolism, high levels of chronic stress in the fish, and, consequently, major susceptibility to opportunistic diseases caused by different pathogens [6]. In fact, infections are problematic in fish farms [1,3,5], resulting in significant production and financial losses of up to 750–1000 million US dollars. [7].

Vibriosis, which is caused by Gram-negative bacteria (like *Vibrio harveyi*, *V. vulnificus*, and *V. anguillarum*), is one of the most common bacterial diseases impacting various marine fish at all stages of growth. Typical signs of disease include lethargic movement, skin ulceration, and hemorrhages. Ultimately, mortality rates of up to 50% can be observed [6]. Another main pathogen is *Lactococcus garvieae*, a Gram-positive bacterium that is present worldwide and causes lactococcosis, a septicaemic–haemorrhagic infection which can result in mortality rates of 80–90%, significantly impacting the economics of fish farming [8]. *L. garvieae* can infect a broad range of farmed and wild fish species, especially when the water temperature exceeds 15 °C, which is common particularly during the summer in the temperate regions. The most relevant affected species are rainbow trout (*Oncorhynchus mykiss*), largemouth black bass (*Micropterus salmoides*), yellowtail amberjack (*Seriola lalandi*), Japanese amberjack (*Seriola quinqueradiata*), the black rockfish (*Sebastes schlegelii*) [8], and also, in recent years, European sea bass (*Dicentrarchus labrax*) [9] and gilthead sea bream (*Sparus aurata*) [8]. The last one, particularly, is the most farmed representative species in the Mediterranean basin and it has considerable economic importance, with a European production of 105,450.84 t live weight (Italy contributing is approximately 7.54%) [8]. Moreover, *Aeromonas salmonicida* causes furunculosis in salmonids [10], and *Photobacterium damselae* subsp. *piscicida* affects important economical Mediterranean species like sea bass and sea bream. Regarding viral pathogens, they can readily affect adult fish under poor environmental conditions or when kept at high densities, and they can be particularly harmful during vulnerable finfish stages, such as larvae and fry, causing significant disease and economic losses in aquaculture [11]. The haemorrhagic septicaemia virus (VHSv), a rhabdovirus belonging to the genus *Novirhabdovirus*, and the infectious pancreatic necrosis virus, (IPNv), from the family *Birnaviridae*, mainly affect salmonids and rainbow trout fish in different environments.

Spring viremia of carp (SVCv), genus *Sprivivirus*, is specific for common carp (Cyprinus carpio) and other cyprinid fishes, while viral necrosis virus (VNNv) is able to infect various marine fish species, including gilthead sea bream and European sea bass [12]. Since the beginning, the aquaculture industry has been using antibiotics to prevent or control epidemics with little regulation, but with the increasing presence of antimicrobial resistance (AMR), it has become important to regulate and limit their use. Therefore, mitigation strategies have been executed, especially in the most developed countries [5,13]. In 2008, over 90% of environmental bacteria isolated in saltwater were resistant to multiple antibiotics, with 20% being resistant to at least one [14].

In this context, antimicrobial peptides (AMPs) are a particularly promising class of molecules that can be considered to fight aquatic pathogens without the negative effects of antibiotics [5]. AMPs are small molecules, constituted by 18–46 amino acids residues, that usually display an amphipathic character and a positive net charge [15]. They are a crucial component of the immune system of all living organisms and have been preserved throughout evolution from prokaryotes to mammals [16]. Finally, AMPs display a broad spectrum of action [17], being active against viruses, bacteria, and parasites, and usually show immunomodulatory properties [18,19]. AMPs carry out their activity using the pathogen (bacterial or virus) membranes as the first target. They cause its destabilization, pore formation, or disintegration, finally resulting in the leakage of cellular contents [16,20]. This mode of action makes it significantly harder for microorganisms to develop resistance [21].

Regarding antiviral effects, AMPs have been shown to have the highest activity against enveloped viruses [22], which contain proteins essential for entry into host cells through the fusion with their cell membrane. In this case, AMPs typically act through membrane-destroying activity and anti-fusion properties. AMPs have already been tested in the food industry and swine and cattle farming and have been shown to boost immunity and enhance production performance [23]. Several studies have also shown that specific peptides exhibit interesting antimicrobial activity against drug-resistant human pathogenic bacteria and fungi [24]. Some peptide formulations, such as daptomycin, have already been approved for human administration and used in the treatment of complicated infections and bacteremia caused by drug-resistant bacteria in a clinical environment, demonstrating their validity as commercial therapies [24].

In this study, we examined the possible application of three peptides, Trematocine (Tmc), Chionodracine (Cnd), and a mutant of this last peptide (Cnd-m3), in the aquaculture sector (see Table 1). Tmc and Cnd are AMPs directly isolated from the Antarctic fish *Trematomus bernacchii* [25] and *Chionodraco hamatus* [26], respectively, while Cnd-m3 was designed directly from the Cnd primary sequence to improve its antimicrobial properties against human pathogens [27]. As a positive charge is fundamental for interactions with the negatively charged membranes of bacteria, within the Cnd sequence, histidines and serines were all replaced by lysines to obtain the Cnd-m3 peptide, and, in this way, the total net charge increased from +2 to +7. CD spectras suggest that this peptide adopts an α-helical conformation upon interaction with synthetic bacterial membranes [27]. These peptides belong to the class of piscidin, a family of amphipathic, α-helical cationic peptides that have been identified in teleost fish [15]. Usually, they show a broad spectrum of action and are effective, for example, against antibiotic-resistant bacterial strains, fungi, and viruses, especially those of aquatic origin [17]. Both Tmc and Cnd showed high antibacterial activity against endemic bacteria from Antarctica [25,26], whereas Cnd-m3 is able to kill human pathogens [27]. For these reasons, we decided to examine the activity of the three peptides against some of the most relevant bacteria and viruses in aquaculture, trying to evaluate their mode of action. Successively, we investigated their haemolytic activity against fish erythrocytes and cytotoxicity versus a continuous embryonic cell line (DLEC) derived from European sea bass. Finally, in light of a possible *in vivo* application, we decided to investigate their immunomodulatory effect.

## 2. Results

### 2.1. Membranolytic Activity of Peptides on Selected Bacteria

**Figure 1:**
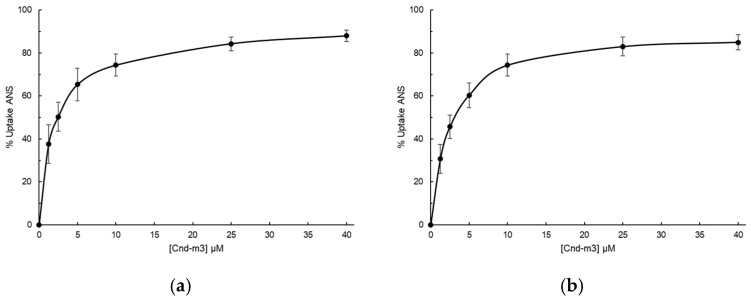
Percentage of ANS uptake of *L. garviae* R (**a**) and *V. harveyi* R (**b**) as a function of Cnd-m3 peptide concentration.

We first decided to investigate the permeabilizing effect of Tmc, Cnd, and Cnd-m3 peptides on the outer membrane of Gram-negative bacterium *Vibrio harveyi* R (reference strain) and on the plasmatic membrane of Gram-positive bacterium *Lactococcus garvieae* R (reference strain). We exploited the specificity of the ANS probe, whose fluorescence is weak in an aqueous medium, but it becomes high in a hydrophobic environment. Hence, if a perturbation/disruption of a cell membrane occurs in the solution, the ANS is able to penetrate into the double lipid bilayer, thus leading to an increase in fluorescence intensity [28]. Both Tmc and Cnd had no effect, and, therefore, only the Cnd-m3 results are presented. In Figure 1 (Panel A and Panel B), the percentage of ANS uptake for the two bacteria upon the addition of increasing amounts of the peptide is shown. The rapid increase in ANS fluorescence indicated that Cnd-m3 perturbs both microorganisms. Regarding *Lactococcus garvieae* R, the 50% of uptake is reached at a concentration of 2.5 µM, whereas about 4 µM is needed for *Vibrio harveyi* R; the same value of 25 µM is enough to obtain 80% of the uptake for both bacteria.

### 2.2. Antibacterial Activity of the Peptides

The *in vitro* MIC and MBC values obtained for the tested bacteria are shown in Table 2. The reference strains were used as the gold standard for the analysis (by using reference strains, the test can be repeated by other laboratories), but we also wanted to evaluate the efficacy and robustness of the results by including more recent, wild-type, and from different origins bacterial strains. The Cnd-m3 peptide gave the best results in terms of MIC (Table 2) against all fish pathogens, except for the field (F) strain of *V. harveyi* (>50 µM) that was resistant to all peptides. Very low MIC values were determined for both the reference (R) and field (F) strains with regard to *P. damselae* sub. *piscicida* (6.25 and 3.125 µM, respectively); good results were also observed for *L. garvieae* R and F and for *V. anguillarum* R and F strains. The Tmc peptide was less active compared to Cnd-m3 for all tested bacteria, whereas Cnd was less or not active in most cases. MBC values (Table 2) confirmed the trend of the MIC results for all tested bacteria, highlighting that Cnd-m3 was the best performer, especially considering *P. damselae* sub. *piscicida*, 6.25 µM for both R and F strains and the *V. harveyi* R strain.

### 2.3. TEM Analysis and Immunoelectron Microscopy

**Figure 2:**
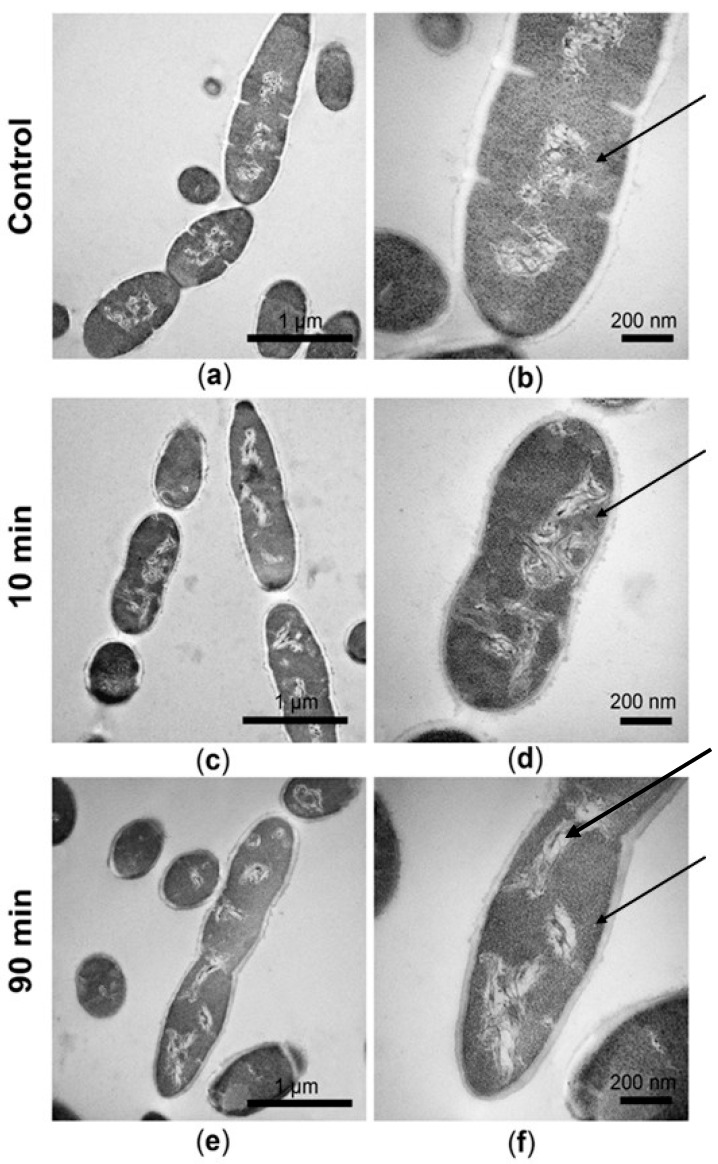
Transmission electron microscopy (TEM) micrographs of *Lactococcus garvieae* R untreated and treated with 5 µM Cnd-m3 for 10 and 90 min. (**a**,**b**) Untreated bacteria (two different magnifications); (**c**,**d**) treated for 10 min (two different magnifications); and (**e**,**f**) treated for 90 min (two different magnifications). The scale is 1 μm for (**a**,**c**,**e**), while the cell details are reported with a scale of 200 nm in (**b**,**d**,**f**). The arrows indicate the difference in the nucleoid region.

To investigate the mechanism of action used by AMPs to kill bacteria, the morphology of the *L. garvieae* R cells treated with a concentration of Cnd-m3 close to 1X MIC (5 μM) was visualized with a TEM analysis. We chose this peptide as it was the most effective in the *in vitro* assays, and we chose this pathogen for its importance on an aquacultural level. Untreated cells (Figure 2a,b) showed an intact surface and dense internal structure, with their cytoplasmic content normally distributed. After a 10 min treatment with the peptide (Figure 2c,d), we observed a change in the structure of the nucleoid region with some sign of membrane perturbation but no clear evidence of cell membrane disruption; successively, after 90 min (Figure 2e,f), the nucleoid region is completely modified compared to the control, and the genetic material of the bacterial cell is deconstructed. Therefore, this is preliminary evidence of the possible way used by the peptide to cause cell bacterial death.

**Figure 3:**
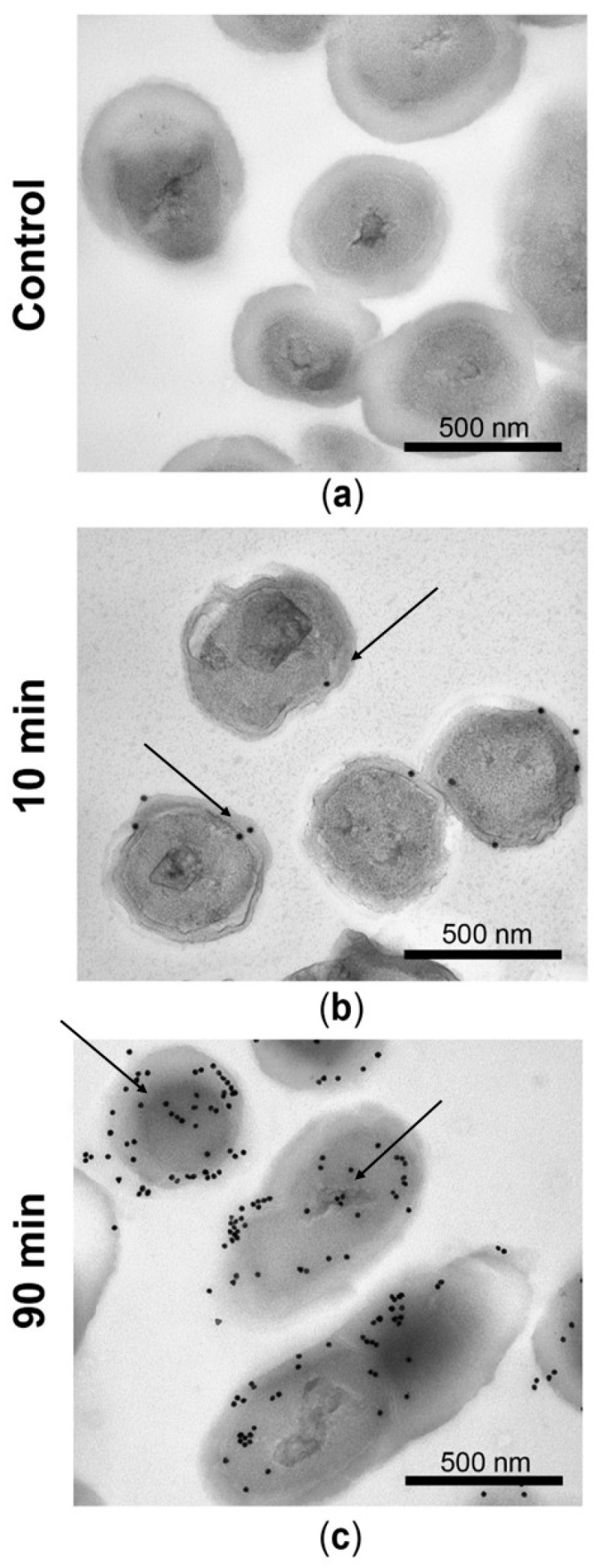
TEM immunogold images of *Lactococcus garvieae* cells incubated with Cnd-m3 and added with a polyclonal antibody able to localize the peptide. (**a**) Bacteria cells treated with the pre-immune antibody serum; (**b**,**c**) bacteria cells after 10 and 90 min treatment with both the peptide and the antibody, respectively. The scale is 500 nm. The arrows indicate the black dot signals of the peptide.

Moreover, to better understand the peptide action on bacterial cells, we performed immunoelectron microscopy (Figure 3) using a polyclonal antibody produced specifically for the peptide that is, therefore, able to localize it. Cnd-m3 (Figure 3b) interacts with the target bacterial cell wall using its positive amino acid residues against the negative exposed charges present on the bacterial cell surface, and it is able to spontaneously traverse the cytoplasmic membrane. Successively (Figure 3c), we visualized it inside the cell, probably targeting the genetic material of the bacteria and causing a consequent microbial cell death. We tried a semi-quantification of the visualized phenomenon and counted the number of gold particles (sign of peptide localization) on the cell membrane and in the cytoplasm after 10 min and 90 min. In the first case, considering 40 casually selected bacterial cells, we have 100 particles on the cell membrane and 10 in the cytoplasm, whereas after 90 min, the values are 270 on the cell membrane and 150 in the cytoplasm, with an increase from 10% to 35% of the peptide inside the bacteria and, therefore, the possible confirmation of an internal target.

### 2.4. Antiviral Activity of the Peptides

We first determined the cytotoxicity of the three peptides (Tmc, Cnd, and Cnd-m3) on the two cell lines used for virus detection. Tmc, investigated at a concentration range from 15 to 5 µM, showed toxicity levels ranging from 50 to 8% against E-11 and from 59 to 23% for EPC, respectively. Cnd concentrations from 25 to 5 µM showed no cytotoxicity against the selected cell lines (EPC and E-11), whereas Cnd-m3, tested from 25 µM to 5 µM, showed light toxicity on E-11 and EPC ranging from 21 to 12%, respectively. According to cytotoxicity results, it was decided to use, in successive experiments of antiviral activity, a concentration of 5 µM for Tmc, 5 and 10 µM for Cnd-m3, and 5 and 15 µM for Cnd.

The peptides’ capability to inhibit viral growth was assessed after incubating the investigated AMPs overnight at 4 °C with the selected viral titres before being inoculated onto the 24 h old grown cells monolayers. The rationale for comparing conditions with and without overnight incubation was to assess whether the peptides present direct virucidal activity or inhibit viral adsorption. On the VNN virus, we tested Tmc at 5 µM, Cnd at 15 µM, and Cnd-m3 at 5 and 10 µM, but no viral inhibition was observed with any of the AMPs used. On enveloped virus VHS, among the three AMPs tested, only Tmc at 5 µM showed viral inhibition, reducing the inoculum titre of 10^2^ (from 10^6.05^ TCID50/mL to 10^4^ TCID50/mL).

For this reason, it was decided to focus on Tmc only, and two more viruses, namely, the SVCV and the IPN, were tested. Tmc showed viral inhibition versus the enveloped virus SVCV, reducing more than 10^2^ the inoculum titre (from 10^5.5^ TCID50/mL to 10^3^ TCID50/mL). To further investigate the mechanism of action of Tmc, the peptide viral inhibition ability against VHS, was compared with or without overnight incubation. Interestingly, the viral inhibition was observed only after the overnight incubation, whereas if the AMP + virus mix was inoculated directly on cells, no reduction in viral titre was observed. Finally, in addition to 4 °C, we tested different overnight incubation temperatures: 15 °C for Tmc + VHSv and 20 °C for Tmc + SVCv. Although we used two different temperatures for overnight incubation (4 °C and 15 °C), the VHSv titre was reduced by 10^2^ in both cases, showing no difference between the investigated conditions. For SVCV, a lighter reduction in the viral titer was observed (around 10^0.5^ more) after overnight incubation at 20 °C compared to 4 °C.

### 2.5. Cytotoxicity of Cnd-m3 and Tmc on DLEC Cell Line

**Figure 4:**
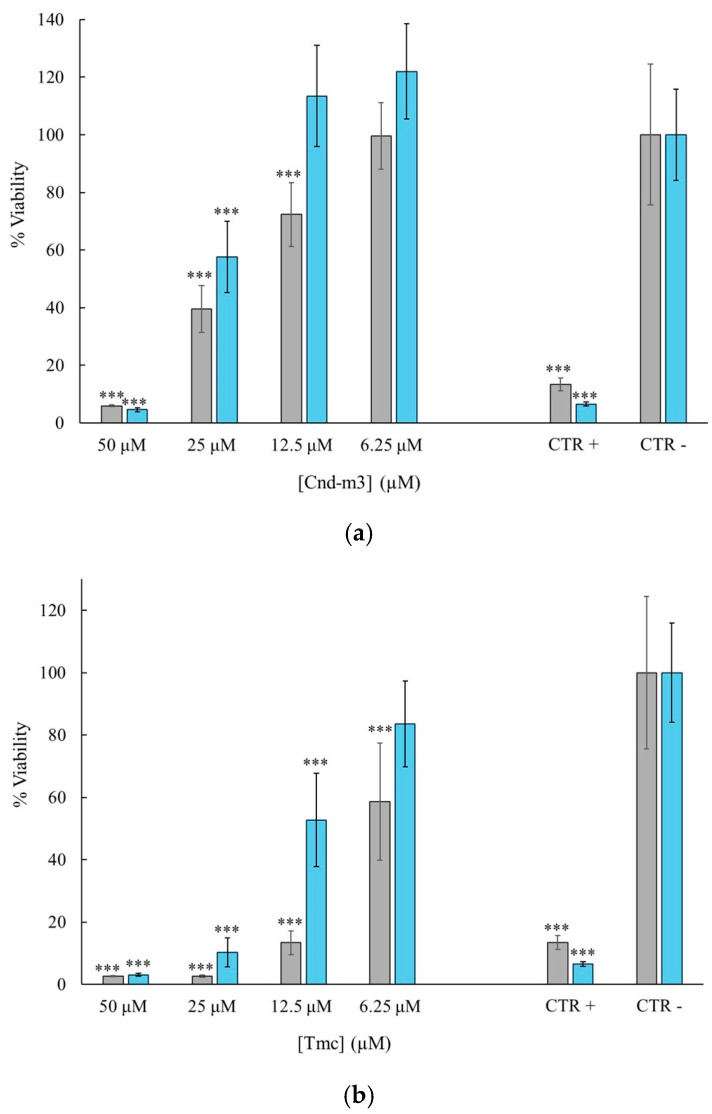
Cytotoxic activity of Cnd-m3 (**a**) and Tmc (**b**) after 6 h (grey) and 12 h (blue) of treatment against a sea bass cell line. Four different concentrations have been tested. The values represent the mean ± SD (N = 6 technical replicates), and the asterisks indicate the significance level with respect to negative control (100% of viability); *** = *p* ≤ 0.0001.

The selectivity of the peptides Cnd-m3 and Tmc have been evaluated by investigating their cytotoxicity against the cell line of an important fish in aquaculture, a sea bass continuous embryonic cell line called DLEC [29], using four different concentrations and two treatment times and comparing the data with the negative control (Figure 4). Cnd-m3 (Figure 4a) showed no cytotoxic activity at 6.25 µM both after 6 and 12 h from the treatment, whereas it was toxic (about 75% viability) at 12.5 µM after 6 h. At higher concentrations, an increase in lethality was observed. Regarding Tmc (Figure 4b), even at the lowest used concentrations, 6.25 µM, a cytotoxic effect was noted (about 60% viability), especially after 6 h from the treatment, whereas after 12 h, the level of toxicity was almost acceptable (about 80% viability).

### 2.6. Haemolytic Activity of Cnd-m3 and Tmc on European Sea Bass Erythrocytes

**Figure 5:**
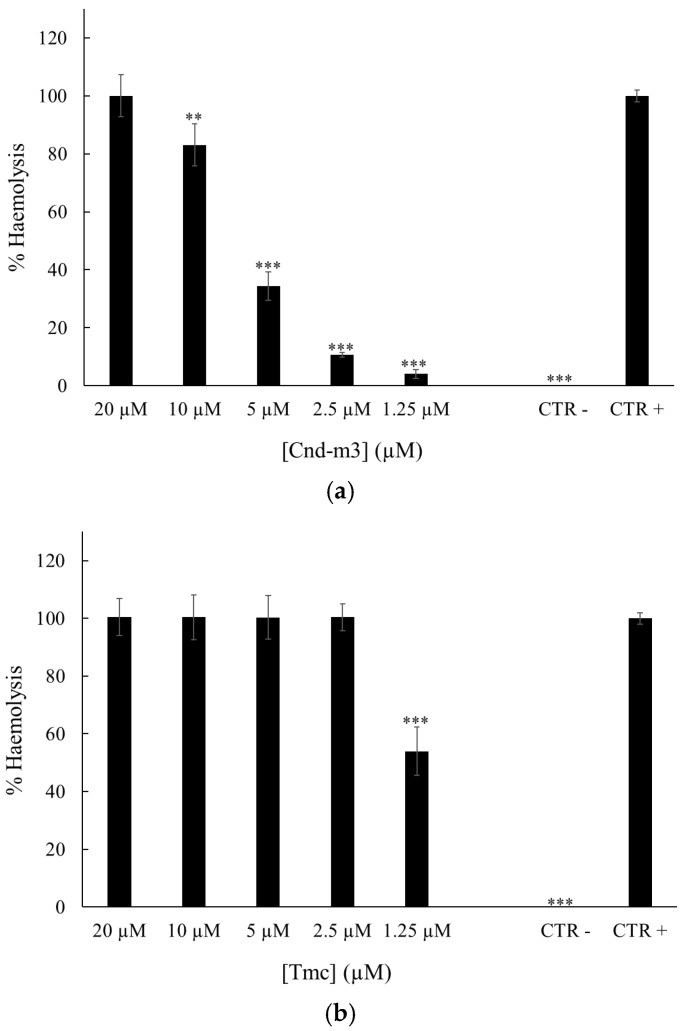
Hemolysis of Cnd-m3 (**a**) and Tmc (**b**) at five different concentrations against sea bass erythrocytes. The values represent the mean ± SD, and the asterisks indicate the significance level with respect to positive control (100% of haemolysis); *** = *p* ≤ 0.0001 and ** = *p* ≤ 0.005.

The hemolytic activity of Cnd-m3 and Tmc was tested against European sea bass erythrocytes (Figure 5). Cnd-m3 showed very low hemolytic activity on sea bass erythrocytes at both 1.25 and 2.5 µM and a slight effect at 5 µM. Tmc already demonstrated 50% of hemolysis at 1.25 µM.

### 2.7. Immunomodulation of Cnd-m3 on DLEC Cell Line

**Figure 6:**
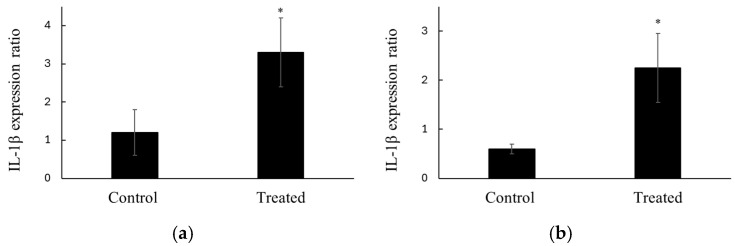
IL-1β expression after DLEC stimulation with 3 µM Cnd-m3 for 6 (**a**) and 24 (**b**) h. The values represent the mean ± SD, and the asterisks indicate the significance level with respect to the control; * = *p* < 0.05.

The immunomodulatory effect of the Cnd-m3 peptide (Figure 6) was studied on the DLEC cell line after 6 and 24 h of *in vitro* stimulation at a concentration of 3 µM. Several inflammatory genes (IL-1β, COX-2, TGF-β, and TNF-α) and a specific AMP from sea bass, dicentracine [30], were investigated: only for IL-1β was a slight up-regulation found at both 6 and 24 h post-treatment (Figure 6a,b).

## 3. Discussion

Aquaculture is among the fastest growing food production sectors [31]. This development is principally due to an increase in global demand and per capita consumption of seafood [3,4], which has increased by more than twofold since 1960, as well as a gradual reduction in the yield of capture fisheries [32], which alone are unable to satisfy dietary needs. In fact, according to a Food and Agriculture Organization of the United Nations (FAO) report, in 2022, global aquatic animal aquaculture production reached 130.9 million metric tons, accounting for more than 50% of total marine food production [33].

Confirming the importance of aquaculture in the global food system, aquatic products are the second most important source of animal protein for humans after milk, contributing about 20% of total animal protein consumption [31].

However, recurrent bacterial and viral infections result in significant financial and productivity losses [6,34], amounting to $6 billion annually, undermining the growth prospects and sustainability of this sector [35], and are expected to increase as temperatures rise due to global warming [36]. To overcome this difficulty, aquaculture needs to use antibiotics. However, these molecules are associated with several disadvantages, including promoting the selection of antibiotic-resistant bacteria and the consequent environmental and safety issues for consumers [34,36,37]. Vaccines are an alternative to antibiotics, but they are not available for many pathogens and/or fish species. Antimicrobial peptides have a broad spectrum of action [17] and could represent a class of valid and ecologically acceptable biomolecules for the treatment of bacterial and viral diseases [38]. This research explored the capacity of two natural peptides, Trematocine and Chionodracine, and a mutant peptide of one of them, Cnd-m3, to interact with some viral and bacterial pathogens related to aquaculture. Regarding the mechanism of action, AMPs act through two mechanisms. The first mechanism involves directly altering the cell membrane by forming small pores or holes [20]. Four main models have been proposed to describe this process: the barrel-stave, the carpet-like, the micellization, and the toroidal pore model [39]. The second mechanism, after an alteration of the membrane, consists of an interaction between AMPs and intracellular targets [39], which leads to the disruption of essential biological processes, such as DNA replication, transcription, and others. In this case, peptides initially bind to the bacterial surface, then translocate into the cell through transient pore formation or internalization mechanisms, such as endocytosis [39]. Interaction primarily occurs through electrostatic forces and hydrophobic interactions, which alter the membrane’s permeability and integrity.

For this reason, we started to evaluate, by ANS fluorescence assay, the ability of the three peptides to interact with and alter the membranes of two bacterial strains of particular concern in aquaculture: *V. harveyi* and *L. garvieae*. The Cnd-m3 peptide showed interesting results, especially against *L. garvieae*, with 50% of ANS uptake already at a concentration of 2.5 µM. We then evaluated the antibacterial activity of the three peptides against five bacterial strains: *V. harveyi*, *V. anguillarum*, *L. garvieae*, *A. salmonicida*, and *P. damselae* sub. *piscicida*. These bacteria can affect a variety of economically important farmed fish species, causing significant financial losses due to their mortality and morbidity [6].

From the data reported in Table 2, it is possible to highlight that the Cnd-m3 peptide gave the best results against all tested bacteria. Regarding the high activity of Cnd-m3 compared to Chionodracine, these results agreed with what we found in previous studies [27]; in fact, Cnd-m3 was designed to increase Chionodracine’s antimicrobial activity and selectivity toward drug-resistant bacteria. Overall, the MIC and MBC values found for Cnd-m3 are close with those observed for other piscidines [40,41]. For example, peptides isolated from European sea bass showed MIC values ranging from 30 to 50 μM against *L. garvieae* [41].

Since Cnd-m3 was identified as the most promising peptide against bacteria, we decided to perform transmission and scanning electron microscopy analyses by treating *L. garvieae* at 5 μM, a concentration near 1X MIC, to obtain insights about its mechanism of action. We chose this bacterium because it is a relevant pathogen in global fish farming [37,42], and it has caused, in recent years, high mortality in European sea bass and gilthead sea bream farms, which are fish species with high commercial importance in the Mediterranean basin. The results obtained suggest that the Cnd-m3 peptide initially interacts with the cell membrane before translocating into the cell, where it exerts its antimicrobial activity by interacting with intracellular targets. However, this preliminary evidence should be confirmed by successive experiments.

Considering the broad spectrum of action of AMPs [43,44,45], and that piscidin often exhibits antiviral activity [46], we decided to investigate whether the three peptides are active against some most significant RNA viruses in aquaculture: two enveloped viruses (VHSv and SVCv), which have a lipid bilayer membrane on the outer surface, and two naked viruses (IPNv and VNNv). Interestingly, only Trematocine at a concentration of 5 μM showed significant antiviral activity against the enveloped viruses VHSv and SVCv, reducing the viral titer by over two logarithms, specifically from 10^6.5^ TCID50/mL to 10^4^ TCID50/mL and from 10^5.5^ TCID50/mL to 10^3^ TCID50/mL, respectively.

Moreover, Trematocine efficacy appeared to be a time-dependent but temperature-independent phenomenon. The viral envelope plays a fundamental role in the infection process, determining interactions with host cells, entry inside them, and, consequently, the replication of genetic materials, and it therefore represents a strategic target for the development of antiviral drugs. The envelope is particularly susceptible to peptides with antiviral activity, which can interact with its structural components and neutralize them. Trematocine is hydrophobic and adopts an α-helical structure when interacting with anionic membranes [25]. These properties facilitate interaction with and affinity for the envelope, thereby enhancing its antiviral efficacy [22,43]. Also, as reported in the literature, hydrophobic α-helical peptides tend to mostly interact with enveloped viruses compared to naked viruses, compromising their integrity and infectious capacity [43]. However, further assays with a large panel of enveloped and non-enveloped viruses should be performed to confirm the interaction between the viral envelope and Trematocine.

Having obtained promising results for the Cnd-m3 and Tmc peptides, we wanted to evaluate their selectivity profile to preliminary explore potential *in vivo* applications in aquaculture. European sea bass is fundamental for the aquaculture economy in the Mediterranean basin, with a production of 7400 tons in 2022, only considering Italy [9]. We therefore assessed the *in vitro* haemolytic and cytotoxic activity of the two peptides against European sea bass erythrocytes and a European sea bass cell line (DLEC), respectively. Overall, Cnd-m3 showed a better compatibility profile, exhibiting low cytotoxic activity and hemolysis at ~5 µM. Trematocine showed higher levels of cytotoxicity, even at the lowest concentrations used, with only 80% viability at 6.25 μM and 60% haemolysis at 1.25 μM. With respect to Cnd-m3, we also determined the selectivity index (SI) against *L. garvieae* R, *V. harveyi* R, and *P. damselae* sub. piscicida R as a ratio between toxic effects (L_50_ versus DLEC cells) and antimicrobial activity (MIC) [44]. The obtained SI value was for all the three bacterial strains, and therefore, it does not reach 10, which indicates desirable selectivity, but it is higher than 3, which is considered the minimum to have a potential applicative interest.

Finally, since it is well known that peptides belonging to the piscidin class showed immunomodulatory effects [15,46], we studied the immunomodulatory properties of the Cnd-m3 peptide on the DLEC cell line after 6 and 24 h of *in vitro* stimulation using a non-toxic and haemolytic concentration (3 µM). Of all the inflammatory genes tested (IL-1β, COX-2, TGF-β, and TNF-α), only IL-1β, a pro-inflammatory cytokine, showed slight up-regulation. This result was in line with the results obtained by Peter Chiou P. and colleagues (2006) [47], as they showed that treatment of the rainbow trout RTS11 macrophage cell line with the pleurocidin peptide determined an increase in the expression of interleukin IL-1β transcripts. Interleukin-1β plays a crucial role in inducing immune responses in fish [48]. It constitutes the primary line of defence against pathogens, instigating inflammatory responses, which include the mobilization of immune cells, such as leucocytes, to the site of infection and the up-regulation of other immune mediators and cytokines [49,50].

Although more evidence should be added, these results suggest that Cnd-m3 could be used not only as an antimicrobial agent against impactful aquaculture bacteria, such as *L. garvieae*, but also possibly as an adjuvant to improve the immunological response of fish, contributing to a better overall immune response, which is fundamental for disease prevention. This is particularly important in the context of intensive farming, where the natural ability of fish to combat disease is compromised by the considerable stress to which they can be subjected [6].

In conclusion, our preliminary studies determined the interesting properties of Cnd-m3 as a potential new “weapon” against fish bacterial targets, with the next step related to its *in vivo* use in aquaculture projects, as already reported for other AMPs, or alone or in combination with vaccination procedures to enhance their action [50]. However, some aspects still need to be studied in detail, such as the effects of AMPs on non-target or beneficial bacteria. Recently, different papers have studied the mechanisms by which AMPs influence host gut microbiota, aiming to contribute to a comprehensive assessment of their advantages as substitutes for antibiotics in fish health and improving aquaculture practices [51,52]. Moreover, for a practical application of AMPs in aquaculture, some issues remain to be solved, like the stability to peptidases and proteases that usually needs to be improved by encapsulating the peptides in liposomes or nanostructures, the relatively high production costs, the potential toxicity versus host cells that needs to be determined for each AMP, and the development of resistance, which is lower compared to that generated by the use of antibiotics but is still possible [16,20].

## 4. Materials and Methods

### 4.1. Peptides

Trematocine (Tmc: FFGHLLRGIVSVGKHIHGLITG), Chionodracine (Cnd: FFGHLYRGITSVVKHVHGLLSG), and mutated Chionodracine (Cnd-m3: WFGKLYRGITKVVKKVKGLLKG) peptides were purchased from CASLO ApS (Lundtofte, Denmark) with a purity > 98%. Peptide concentrations were determined by absorption spectroscopy at 280 nm before each analysis.

### 4.2. Bacterial Strains

*Lactococcus garvieae* (Gram-positive) reference (R, culture colony n. DSMZ 6783, ATCC 6783) and field (F, from *Onchorhynchus mykiss*) strains, *Vibrio harveyi* (Gram-negative) R (culture colony n. LMG 4044, ATCC 14126) and F strains (from *Dicentrarchus labrax*), *Aeromonas salmonicida* (Gram-negative) R (culture colony n. NCIMB 1102, ATCC 33658) and F strains (from *Onchorhynchus mykiss*), *Photobacterium damselae* subsp. *piscicida* (Gram-negative) R (culture colony n. DSMZ 22834, ATCC 51736) and F strains (from *Dicentrarchus labrax*), and *Vibrio anguillarum* 01 (Gram-negative) R (culture colony n. LMG 10861, ATCC 43305) and F strains (from *Dicentrarchus labrax*) were comprised in the collection of Italian National Reference Centre for Fish, Shellfish and Molluscs Diseases (Istituto Zooprofilattico Sperimentale delle Venezie, Legnaro, Padova, Italy).

### 4.3. Outer Membrane Permeability Essay

The fluorescent probe 1-aminonaphtalene-8-sulfonic acid (ANS, Merck, Saint Louis, MO, USA) uptake was used to investigate the interaction of Tmc, Cnd, and Cnd-m3 peptides with the bacterial cell wall. Specifically, the Gram-negative *V. harveyi* and the Gram-positive *L. garvieae* bacteria were grown at 22 °C in MAB and TSB + YE medium (Merck, Saint Louis, MO, USA), respectively. Successively, the cell suspensions were maintained in saline phosphate buffer (PBS) at an OD_600_ of 0.6. Different concentrations of the peptides (ranging from 0 to 40 μM) were added in 700 mL of cell suspension containing 25 μM ANS. Fluorescence spectra were recorded from 400 nm to 600 nm with a PerkinElmer fluorometer LS 55 in steady state at room temperature, using an excitation wavelength of 360 nm and excitation/emission band pass of 5 nm. The cell membrane integrity was quantified measuring the fluorescence intensity [27] using the equation:

(1) $$\%UptakeANS=\frac{\left(F-F_{0}\right)}{F}$$

where F is the fluorescence of ANS observed at a given peptide concentration, and F_0_ is the fluorescence of ANS in the absence of peptides.

### 4.4. Determination of Minimum Inhibitory Concentration (MIC) and Minimum Bactericidal Concentration (MBC) for the Selected Bacterial Strains

Minimum inhibitory concentrations were determined using serial 2-fold dilutions in PBS of the peptides in 96-well microplates. Bacterial cultures were suspended in PBS (0.5 McFarland standard) and then diluted in Cation-adjusted Mueller–Hinton Broth (CAMHB) (Merck, Saint Louis, MO, USA) to a final concentration of 10^5^ CFU/mL. Each well contained 100 µL of diluted AMP and 100 µL of bacterial suspension. The final concentration of the peptides ranged from 0.024 to 50 µM. Plates were read after 24 h of incubation at 22 °C. The MIC value corresponds to the lowest concentration of the peptide at which bacterial growth is visually inhibited. To obtain the minimum bactericidal concentration (MBC) value, 1 µL of each analyzed sample was streaked onto a blood agar plate using a sterile 1 µL loop. The MBC value is the lowest peptide concentration capable of killing the bacterium under investigation; this concentration is determined observing bacterial growth on a plate after 24 h of incubation at 22 °C.

### 4.5. TEM Analysis and Immunoelectron Microscopy

*Lactococcus garvieae* R grown at mid-logarithmic phase (5 × 10^7^ CFU/mL) was treated with 5 µM (close to MIC value) Cnd-m3 for 10 min and 90 min with shaking or was untreated (control). At the end of the incubation time, samples were centrifuged (5000 rpm for 5 min), and the supernatant was discarded. They were collected and fixed as described before [53]. After rinsing in the same buffer for 10 min, samples were dehydrated in a graded ethanol series and embedded in medium-grade LR white resin. Ultrathin sections were realized as described before [53].

For immunogold staining (IGS), the sections, obtained as above, were incubated overnight in a moist chamber with the polyclonal antibody Peps2 [53] diluted 1:1000 in TRIS-HCl buffer, with pH 7.6. The procedure used for staining has been described before [53]. Pre-immune serum substituted the primary antibody in control sections.

Sections were stained with uranyl acetate and lead citrate and observed with a Jeol JEM EX II transmission electron microscope at 100 kV.

### 4.6. Fish Cell Lines Viability Assay

Two cell lines were selected for the virus detection: *Epithelioma papulosum cyprinid* (EPC, ATCC Catalogue No. CRL-2872) [54] and E-11 (ECACC Catalogue No. 01110916, a clone of the striped snakehead fry (SNN-1)) [55]. Fish cell viability assay was performed using 3-(4,5-dimethylthiazol-2-yl)-2,5-diphenyl tetrazolium bromide (MTT) from Merck (Saint Louis, MO, USA), which was dissolved in Minimum Essential Medium (MEM, MerckSaint Louis, MO, USA) at a concentration of 1 mg/mL and filtered with a 0.22 µm filter.

Cells to be tested were plated in a 96-multiwell plate, considering, at least, duplication for each condition, and the plate was always incubated at a temperature dependent on the specific value requested for the tested fish cell lines (namely, 15 °C and 25 °C for E-11). Approximately 24 h after plating, when the cells formed a monolayer, the culture medium was removed and fresh medium with serial dilution of AMPs was added. Approximately 24 h later, the medium with AMPs was removed, 100 µL of MTT was added to each well, and the plate was incubated for 4 h at the usual temperature. Successively, the MTT medium was removed, and 100 µL of DMSO (Sigma-Aldrich, St. Louis, MO, USA) was added to each well in order to dissolve the formazan crystals present at the bottom of the wells. After 5 min of incubation at room temperature in the dark, the absorbance was read at 570 nm with a Tecan Sunrise spectrophotometer. Percentage of cytotoxicity was calculated with the following equation:

(2) $$\%Cellviability=100-\frac{\left(Control-Sample\right)}{Control}\%$$

### 4.7. Viral Inhibition Test

The selected fish viruses were virus hemorrhagic septicemia (VHSv) genotype 1aI, spring viremia of carp (SVCv), infectious pancreatic necrosis (IPNv), and betanodavirus (VNNv) genotype RGNNV. All of them are RNA viruses: two are enveloped (namely, VHSv and SVCv), two are naked (IPNv and VNNv), and all of them cause systemic diseases in farmed fish species. The cell lines were plated in a 96-multiwell plate in order to obtain a confluent monolayer after 24 h. The determined concentration of the three different AMPs (resulting from the MTT test) was separately mixed with a serial dilution of the virus of interest and inoculated on the cells after overnight incubation at 4 °C, 15 °C, or 20 °C (depending on the temperature tolerance of the investigated virus). The plates were incubated at a temperature dependent on the fish cell lines for 7 days and constantly monitored to detect cytopathic effect (ECP). The reduction in viral titer caused by the treatment with AMPs was calculated as the difference, expressed in TCDI50/mL, between treated and control samples. Eventually, the test was repeated without the overnight incubation of peptides and viruses in order to further investigate the AMPs’ mechanism of action.

### 4.8. Cytotoxicity Assays on DLEC Cell Line

The cytotoxicity of Trematocine and Cnd-m3 peptides was tested on a fish cell line obtained from *Dicentrarchus labrax* embryos named DLEC. The cells were maintained at 22 °C in FBS-free L-15 medium and placed on 96-well microplates (Perkin-Elmer, Waltham, MA, USA) at a density of 10^3^ cells per well in a volume of 100 μL overnight. Then, four dilutions of each peptide (from 6.25 μM to 50 μM) were added to the cells and maintained for 6 h and 12 h. The negative control is formed by cells grown in normal medium plus an equivalent amount of water (peptide solvent), while NaN_3_ 0.5% *v*/*v* was inserted in the positive control. The cytotoxicity was determined by measuring the intracellular adenosine triphosphate (ATP) levels using the luciferase-based ATPlite assay (PerkinElmer, Waltham, MA, USA), according to the manufacturer’s instructions with a microplate luminometer (Victor II PerkinElmer) [56]. Six technical replicates per each dilution were performed. Cell viability values were expressed as the mean ±SD and calculated as the percentage values of the treated samples with respect to the negative control.

### 4.9. Hemolytic Activity of Tmc and Cnd-m3

Sea bass (*Dicentrarchus labrax*) blood was collected with a heparinized syringe from healthy fish reared in an experimental aquarium (National Reference Centre for Fish, Shellfish and Molluscs Diseases, Istituto Zooprofilattico Sperimentale delle Venezie, Legnaro, Padova, Italy, blood collection authorization n° 529/2020-PR on 26 May 2020), and the red blood cells were obtained as follows:

(i) Blood was diluted 1:5 in HBSS 1× (Corning, Biosigma, Venezia, Italy), and 5 mL of this diluted solution were gently layered onto 5 mL of Ficoll-Paque™ (GE Healthcare, Milano, Italy) and centrifuged at 2000 rpm for 30 min at 4 °C.
(ii) The supernatant was eliminated, and the red blood cells collected from the pellet were washed twice in 50 mL of HBSS and centrifuged at 1800 rpm for 10 min at 10 °C. The final pellet of red blood cells was resuspended in 10 mL of PBS 0.01 M and counted in a Burker chamber.
(iii) Red blood cells were plated at a concentration of 2.5 × 10^6^ per well in a 96-well plate, considering triplicates for each condition.
(iv) Peptides were added to the wells at different selected concentrations (1.25, 2.5, 5, 10 and 20 μM), while 2% Triton-X (Merck, Saint Louis, MO, USA) diluted in PBS 0.01 M was used as a positive control for red blood cell lysis.
(v) The plate was incubated for 2 h at 20 °C and spun for 3 min at 1200 rpm at 10 °C.
(vi) The supernatant was then transferred to a new plate, and the absorbance was read at 492 nm with a Tecan Sunrise spectrophotometer. The haemolytic degree was determined using the following relation [57].

(3) $$\%Haemolysis=\frac{\left({Abs}_{testgroup}-{Abs}_{negativecontrol}\right)}{\left({Abs}_{positivecontrol}-{Abs}_{negativecontrol}\right)}\%$$

### 4.10. Immunomodulatory Activity of Cnd-m3

The immunomodulatory activity of Cnd-m3 peptide was evaluated on DLEC cell line cultured as described in Section 4.8. Three 50 mL flasks with 5 × 10^6^ DLEC cells were stimulated with 3 µM peptide for 6 and 24 h. Total RNA was isolated from each DLEC flask and stimulated separately with TRIsure (Bioline, London, UK) following manufacturer’s instructions, resuspended in DEPC-treated water, and used for real-time quantitative PCR without pooling the samples coming from the different flasks. Controls for the presence of DNA contamination were performed with RT-PCR using β-actin primers that bracket an intron (Table 3). Reverse transcription and real-time PCR was performed as described before [25]. Specific PCR primers were designed for the amplification of about 200 bp products from IL-1β, COX-2, TGF-β, TNF-α, and dicentracine (Table 3), whereas ribosomal RNA 18S and b-actin were tested as house-keeping genes (Table 3). Triplicate reactions were performed for each template cDNA, and the template was replaced with water in all blank control reactions. The analysis was carried out as described before [25] using ribosomal RNA 18 S as the normalizer target and time 0 as the reference transcript (control used like a calibrator). The results are expressed as the mean ± SD, and the differences from the controls have been considered significant if *p* < 0.05 using a statistical analysis performed by one-way ANOVA followed by the Bonferroni test.

## Figures and Tables

**Table 1:** Different physico-chemical characteristics of the tested AMPs.

Peptide	Sequence	Net Charge	Molecular Weight	Hydrophobic Moment	Amphiphilicity Index	Secondary Structure
Chionodracine (Cnd)	FFGHLYRGITSVVKHVHGLLSG	+2	2424.83	1.37	0.71	α-helix
Chionodracine mutant 3 (Cnd-m3)	WFGKLYRGITKVVKKVKGLLKG	+7	2519.16	1.61	1.66	α-helix
Trematocine (Tmc)	FFGHLLRGIVSVGKHIHGLITG	+2	2358.82	1.29	0.48	α-helix

**Table 2:** MIC and MBC values (µM) for the different tested bacterial species. R = reference. F = field. The minimum value for each pathogen is in bold. Concentration range: 0.024–50 µM.

Bacterial Strains	Cnd	Cnd-m3	Tmc
*L. garvieae* R	>50/>50	**6.25/25**	12.5/50
*L. garvieae* F	>50/>50	**12.5/50**	25/**50**
*V. harveyi* R	25/50	**6.25/6.25**	25/25
*V. harveyi* F	**>50/>50**	**>50/>50**	**>50/>50**
*A. salmonicida* R	>50/>50	**25/25**	**25**/50
*A. salmonicida* F	>50/>50	**12.5/12.5**	25/25
*P. damselae* sub. *piscicida* R	25/25	**6.25/6.25**	12.5/25
*P. damselae* sub. *piscicida* F	25/25	**3.125/6.25**	6.25/12.5
*V. anguillarum* R	50/50	**12.5/12.5**	25/25
*V. anguillarum* F	50/>50	**12.5/50**	50/**50**

**Table 3:** List of primers used in RT-PCR.

GENE	PRIMERS (Forward and Reverse)	Accession Number
COX-2	5′-CATTCTTTGCCCAGCACTTCACC-3′ 5′-AGCTTGCCATCCTTGAAGAGTC-3′	AJ630649
TNF-α	5′-CGCAGCACTTTGCTTCG-3′ 5′-TCGTCTTCATCATAGCTACC-3′	DQ200910
TGF-β	5′-GACCTGGGATGGAAGTGG-3′ 5′-CAGCTGCTCACCTTGTG-3′	AM421619
Dicentracine	5′-CTTTCTTGTGCTGTCGATGGT-3′ 5′-AAGCTGCGCGCTCGC-3′	AY303949
IL-1β	5′-GGTGGACAAAGCCAGTC-3′ 5′-CGATGTTGAAGGCTCGG-3′	AJ331925
β-actin	5′-ATGTACGTTGCCATCC-3′ 5′-GAGATGCCACGCTCTC-3′	AJ493428
rRNA 18S	5′-CCAACGAGCTGCTGACC-3′ 5′-CCGTTACCCGTGGTCC-3′	AY831388

## Data Availability

Data supporting the findings of this study are available from the corresponding author upon request.
